# Reply to Sidiropoulos, K.; Tsikopoulos, K. Comment on “Oldrini et al. PHILOS Synthesis for Proximal Humerus Fractures Has High Complications and Reintervention Rates: A Systematic Review and Meta-Analysis. *Life* 2022, *12*, 311”

**DOI:** 10.3390/life12081282

**Published:** 2022-08-22

**Authors:** Lorenzo Massimo Oldrini, Pietro Feltri, Jacopo Albanese, Francesco Marbach, Giuseppe Filardo, Christian Candrian

**Affiliations:** 1 Service of Orthopaedics and Traumatology, Department of Surgery, EOC, 6900 Lugano, Switzerland; 2 Orthopaedics and Traumatology Clinic, EOC, 6900 Lugano, Switzerland; 3 Applied and Translational Research Center (ATRc), IRCCS Istituto Ortopedico Rizzoli, 40136 Bologna, Italy; 4 Faculty of Biomedical Sciences, USI-Università della Svizzera Italiana, 6900 Lugano, Switzerland

Thank you for the opportunity to respond to the commentary [1] on our recent article “PHILOS Synthesis for Proximal Humerus Fractures Has High Complications and Reintervention Rates: A Systematic Review and Meta-Analysis” [2]. We read the commentary with much interest, and we are happy to address all the issues posed. The main aspect we would like to underline is that most of the comments can be simply explained by the fact that the authors of the letter have misinterpreted the focus and message of our systematic review and meta-analysis. The article is based on the premise that the PHILOS plate was developed to improve the success of surgical treatment of proximal humerus fractures in terms of function, number and severity of complications, and reintervention, compared with previous commercially available plates. This was clearly stated in the introductory chapter of the meta-analysis, which states “To overcome these problems and increase patients’ functional outcomes, the AO/ASIF group has developed an anatomic plate design: the Proximal Humeral Internal Locking System (PHILOS) plate”. However, no study had documented the literature evidence on the incidence of complications of the PHILOS plate, which was the focus and provides the added value of this article.

The meta-analysis was able to find a large number of studies that provided important evidence for the purpose of our study. All studies were described and reported in detail in the table, including author, year of publication, study design, and all the major demographic and treatment variables documented, and the references can be found in the supplementary material (for further information, see Table S1).

The commentator points out that some studies do not have sufficient follow-up time. To properly document the complication rate after surgery, we opted to include studies with sufficient follow-up, meaning we had an evaluation encompassing the entire period after surgery until results were stabilized. We believe this aim was reached by our analysis, and also in the studies mentioned in the letter. For example, the articles by Moonot et al., Trepat et al., and Xue et al. reported data of final results up to 24 months. Olerud et al. also specified that all of his patients underwent follow-up at 24 months. The other two studies of Aliudin et al. and George et al. also considered the follow-up sufficient to properly evaluate stable results in terms of complication and final outcome. In any case, these two articles include only 55 patients with a complication rate of 5.45% (3/55 patients) and comprise only 1.32% (55/4165 patients) of the total number of patients analysed. Thus, following the suggestion to exclude these studies, the complication rate would be minimally affected. Moreover, it would be affected in the opposite direction of what was suggested by the letter, leading to an overall even higher percentage of complications. Thus, rather than overestimating the outcome, overall, the article selection and the analysis was conservative, and still able to prove important points on the presence of complications after PHILOS plate implantation.

We confirm this article is based on the study of standard PHILOS plate, and as stated in the exclusion criteria, “long PHILOS […] were also exclusion criteria.” Accordingly, we eliminated all articles that used long PHILOS, along with articles in which PHILOS plates were used for diaphysis fractures. In addition, Table 1 of the main text, column 3 shows that the study design is reported in the “Data Extraction” chapter.

Another misinterpreted aspect is the effect of the quality of the studies on the outcome of the meta-analysis. To characterize the studies, we used the Downs and Black tool, which showed overall low scores. However, this may mainly affect studies focused on the outcome, where the definition of treatment potential is better documented by high-level RCTs. This is not as important when you evaluate the complication rate, where the quality is important, of course, but studies with a lower design such as case series can also provide useful information. Since the purpose of our study is to assess the rate of complications following proximal humerus fractures treated with PHILOS plate, an outcome not influenced by the study design, the low RoB score likely only minimally affects the outcome of the analysis. As for the statistical analysis, it would certainly have been interesting for us to be able to conduct more detailed sub-analyses. However, as the author points out, the quality of the literature was limited; thus, further subgroup analyses would have been inappropriate and at risk of misleading conclusions, which is why the analysis was focused on the main aspects. Finally, we agree that different subgroups can have different risks, as in any field of medicine, and we underlined this as well. Some patients may have, for example, a higher risk of AVN. Even though, as reported by Ayyas et al. [3], not all AVNs require the need for surgical re-intervention, which is reserved mainly for the advanced stages of AVNs, they still remain the main complications for which surgical reintervention is required. This is supported by the most recent meta-analyses, such as the ones by Kavuri et al. [4] and Sproul et al. [5]. While we did not affirm that AVN is caused by PHILOS plating, it is important to understand the frequency and impact of this and other complications. The meta-analysis was performed for this purpose by summarizing a large body of literature, offering a clear representation of the complications and reinterventions documented when using the PHILOS plate. This will help physicians and patients have more realistic expectations of the possible outcome of this surgery and could serve as a reference to the field, starting from these findings, for further efforts to improve the treatment of the challenging proximal humeral fractures.

## Supplementary Materials

The following supporting information can be downloaded at: https://www.mdpi.com/article/10.3390/life12081282/s1, Table S1: Details of the included studies; Pt. (patients), M (male), F (female), DS (delto-split), (DP delto-pectoral).
