# Supplementary material for: Reply to Sidiropoulos, K.; Tsikopoulos, K. Comment on “Oldrini et al. PHILOS Synthesis for Proximal Humerus Fractures Has High Complications and Reintervention Rates: A Systematic Review and Meta-Analysis. Life 2022, 12, 311”
Source: Life (Basel). 2022 Aug 22;12(8):1282. doi: 10.3390/life12081282 (PMC9410006; doi:10.3390/life12081282)
Supplement: Supplementary file 1 [file life-12-01282-s001.zip › life-1859805-supplementary.pdf]

Table S1. Details of the included studies; Pt. (patients), M (male), F (female), DS (delto-split), (DP delto-pectoral).

First Author, Year	Country	Comparative	Pt. (M-F)	Age	Neer Classification			Surgical approach
					N2	N3	N4	
Acklin et al, 2009[1]	Switzerland	NO	29 (9–20)	64	N/A	N/A	N/A	DS
Acklin et al, 2013[2]	Switzerland	NO	97 (N/A)	62	N/A	N/A	N/A	DS
Aggarwal et al, 2010[3]	India	NO	47 (27–20)	58.5	11	22	14	DP
Aliuddin et al, 2016[4]	Pakistan	NO	20 (12–8)	40	4	10	6	DP
Bachelier et al, 2014[5]	Germany	NO	50 (20–30)	62.7	15	18	17	DS
Bandalovic et al, 2014[6]	Croatia	NO	67 (N/A)	N/A	N/A	N/A	N/A	DP/DS
Beeres et al, 2017[7]	Switzerland	YES	282 (85–197)	64	58	153	74	DP/DS
Bhayana et al, 2021[8]	India	YES	84 (45–39)	45	0	40	44	DP/DS
Björkenheim et al, 2004[9]	Finland	NO	72 (28–44)	67	38	22	12	DP
Boesmueller et al, 2016[10]	Austria	NO	154 (61–93)	55.8	41	71	42	DP
Borer et al, 2017[11]	Switzerland	YES	62 (16–46)	64	18	4	10	DP/DS
Boudard et al, 2014[12]	France	YES	33 (19–14)	49.6	0	21	12	DP
Bu et al, 2021[13]	China	YES	48 (17–31)	66.3	28	13	7	DP
Buchmann et al, 2021[14]	Switzerland	YES	198 (75–123)	64.3	N/A	N/A	N/A	DP/DS
Cai et al, 2012[15]	China	YES	12 (1-11)	72.4	0	0	12	DP
Caliskan et al, 2019[16]	Turkey	YES	45 (18–27)	53.2	11	21	13	DS
Cha et al, 2017[17]	South Korea	YES	32 (8–24)	67.8	8	21	3	DP
Chen et al, 2019[18]	China	YES	112 (37–75)	64.29	52	60	0	DP
Chen et al, 2020[19]	Taiwan	YES	35 (13–22)	56.1	12	17	6	DP
Cho et al, 2017[20]	South Korea	NO	39 (12–27)	59	14	22	3	DP
Cohen et al, 2009[21]	Brazil	NO	26 (12–14)	57	7	10	7	DP
Davids et al, 2020[22]	USA	YES	75 (N/A)	59.9	40	35	0	DP
Doshi et al, 2017[23]	India	NO	53 (24–29)	54.3	19	17	11	DP
Erasmo et al, 2014[24]	Italy	NO	81 (39–42)	56	7	40	35	DP
Falez et al, 2019[25]	Italy	NO	76 (26–50)	68.5	3	35	38	DS
Faraj et al, 2011[26]	Netherlands	YES	37 (N/A)	N/A	N/A	N/A	N/A	DS
Fattoretto et al, 2016[27]	Italy	NO	55 (17–38)	63.4	0	16	39	DP/DS
Fazal et al, 2009[28]	UK	NO	27 (6–21)	56	13	12	2	DP
Fraser et al, 2020[29]	Norway	YES	60 (8–52)	74.7	0	29	31	DP
Geiger et al, 2010[30]	Germany	NO	28 (8–20)	60.7	8	12	8	DP
George et al, 2021[31]	India	NO	35 (25–10)	52	12	21	14	DP
Gonc et al, 2017[32]	Turkey	NO	31 (12–19)	58.4	4	14	13	DS
GraC.I.telli et al, 2013[33]	Brazil	NO	40 (12–28)	61.8	16	22	2	DP
GraC.I.telli et al, 2016[34]	Brazil	YES	33 (8–25)	66.4	16	17	0	DP
Handschin et al, 2008[35]	Switzerland	NO	31 (11–20)	62	8	13	10	DP
Hengg et al, 2019[36]	Austria	YES	34 (5–29)	76	5	17	12	DP

Jaura et al, 2014[37]	India	YES	30 (20–10)	65	12	14	4	DP
Klitscher et al, 2008[38]	Germany	NO	30 (11–19)	59	2	16	12	DP
Koukakis et al, 2006[39]	Greece	NO	20 (8–12)	61.7	5	11	4	DP
Kumar et al, 2014[40]	India	NO	51 (35–16)	38	8	15	23	DP
Launonen et al, 2019[41]	UK	YES	44 (3–41)	82	44	0	0	N/A
Lee et al, 2017[42]	South Korea	YES	31 (11–20)	58.6	31	0	0	N/A
Leonard et al, 2009[43]	Ireland	NO	32 (9–23)	61.6	N/A	N/A	N/A	DP
Lorenz et al, 2020[44]	Austria	YES	31 (N/A)	59	0	12	19	DP
LuC.I.ani et al, 2020[45]	Italy	YES	26 (3–23)	73	0	9	15	DP
Martinez et al, 2009[46]	Spain	NO	58 (31–27)	61	0	33	25	DP
MatejC.I.c et al, 2013[47]	Croatia	NO	59 (9–50)	70.5	0	32	27	DP
Miyazaki et al, 2012[48]	Brazil	NO	56 (19–37)	62	13	28	8	DP
Monteiro et al, 2011	Brazil	NO	33 (14–19)	57	17	13	4	DP
Moonot et al, 2007[49]	UK	NO	32 (9–23)	59.9	0	20	12	DP
Norouzi et al, 2012[50]	Iran	NO	37 (27–10)	50.1	13	20	4	N/A
Ockert et al, 2014[51]	Germany	NO	43 (12–31)	58.2	N/A	N/A	N/A	DP
Oh et al, 2015[52]	Germany	NO	26 (6–20)	67	0	17	9	DS
Olerud et al, 2010[53]	Sweden	NO	50 (10–40)	75	50	0	0	DP
Ortmaier et al, 2015[54]	Austria	YES	30 (13–17)	31.3	0	10	20	N/A
Papadopoulos et al, 2009[55]	Greece	NO	29 (12–17)	62.3	0	22	7	DP
Parmaksizoglu et al, 2010[56]	Turkey	NO	32 (10–22)	63	0	12	20	DP
Plath et al, 2019[57]	Germany	YES	32 (7–25)	77.1	4	24	4	DP/DS
Robinson et al, 2010[58]	Scotland	NO	47 (21–26)	57	27	12	8	DS
Seo et al, 2020[59]	South Korea	NO	27 (12–15)	53	5	14	8	DP
Setaro et al, 2020[60]	Italy	YES	64 (N/A)	61.5	37	27	0	DP
Shahid et al, 2008[61]	UK	NO	41 (9–32)	N/A	11	11	19	DP
Shi et al, 2011[62]	China	NO	43 (15–28)	68.7	10	21	12	DP
Shin et al, 2021[63]	South Korea	NO	56 (12–44)	74.3	21	27	8	DP
Siebenbürger et al, 2019[64]	Germany	YES	55 (12–43)	76.6	20	22	13	DP
Sohn et al, 2017[65]	South Korea	YES	90 (N/A)	61.8	35	44	11	DP/DS
Spross et al, 2012[66]	Switzerland	YES	22 (4–18)	75	N/A	N/A	N/A	DP
Spross et al, 2012[67]	Switzerland	NO	294 (71–223)	72.9	N/A	N/A	N/A	DP
Trepat et al, 2012	Spain	YES	11 (3–8)	68.3	11	0	0	DP
Urda et al, 2012[68]	Spain	NO	15 (3–12)	71	15	0	0	DP
Vijayvargiya et al, 2016[69]	India	NO	26 (19–7)	46	5	12	9	DS
Voigt et al, 2011[70]	Germany	YES	31 (N/A)	72	0	27	4	DP
Wang et al, 2019[71]	China	YES	46 (13–33)	72.5	0	0	46	DP
Xue et al, 2018[72]	China	YES	43 (N/A)	57	43	0	0	DS
Zeng et al, 2018[73]	China	YES	181 (64–117)	57.4	78	75	28	DP
Zhao et al, 2019[74]	China	YES	21 (12–9)	69	0	15	6	DP

1. Acklin, Y.P., et al., *Minimal Invasive PHILOS(®)-Plate Osteosynthesis in Proximal Humeral Fractures.* Eur J Trauma Emerg Surg, 2009. **35**(1): p. 35-9.
2. Acklin, Y.P., K. Stoffel, and C. Sommer, *A prospective analysis of the functional and radiological outcomes of minimally invasive plating in proximal humerus fractures.* Injury, 2013. **44**(4): p. 456-60.
3. Aggarwal, S., et al., *Displaced proximal humeral fractures: an Indian experience with locking plates.* J Orthop Surg Res, 2010. **5**: p. 60.
4. Aliuddin, A.M., et al., *Functional Outcome Of Proximal Humeral Fractures Treated With Philos Plate In Adults.* J Ayub Med Coll Abbottabad, 2016. **28**(2): p. 337-340.
5. Bachelier, F., et al., *Treatment for displaced proximal humerus fractures: comparison of interlocking plate fixation versus minimal invasive techniques.* Eur J Orthop Surg Traumatol, 2014. **24**(5): p. 707-14.
6. Bandalović, A., et al., *The Results of Internal Fixation of Proximal Humeral Osteoporotic Fractures with PHILOS Locking Plate.* Psychiatr Danub, 2014. **26 Suppl 2**: p. 376-81.
7. Beeres, F.J.P., et al., *Plate fixation of the proximal humerus: an international multicentre comparative study of postoperative complications.* Arch Orthop Trauma Surg, 2017. **137**(12): p. 1685-1692.
8. Bhayana, H., et al., *Outcomes of plate osteosynthesis for displaced 3-part and 4-part proximal humerus fractures with deltopectoral vs. deltoid split approach.* Eur J Trauma Emerg Surg, 2021.
9. Björkenheim, J.M., J. Pajarinen, and V. Savolainen, *Internal fixation of proximal humeral fractures with a locking compression plate: a retrospective evaluation of 72 patients followed for a minimum of 1 year.* Acta Orthop Scand, 2004. **75**(6): p. 741-5.
10. Boesmueller, S., et al., *Risk factors for humeral head necrosis and non-union after plating in proximal humeral fractures.* Injury, 2016. **47**(2): p. 350-5.
11. Borer, J., et al., *Mid-term results of minimally invasive deltoid-split versus standard open deltopectoral approach for PHILOS™ (proximal humeral internal locking system) osteosynthesis in proximal humeral fractures.* Eur J Trauma Emerg Surg, 2020. **46**(4): p. 825-834.
12. Boudard, G., et al., *Locking plate fixation versus antegrade nailing of 3- and 4-part proximal humerus fractures in patients without osteoporosis. Comparative retrospective study of 63 cases.* Orthop Traumatol Surg Res, 2014. **100**(8): p. 917-24.
13. Bu, G., et al., *MutiLoc Nail Versus Philos Plate in Treating Proximal Humeral Fractures: A Retrospective Study Among the Alderly.* Geriatr Orthop Surg Rehabil, 2021. **12**: p. 21514593211043961.
14. Buchmann, L., et al., *Proximal humerus fractures (PHFs): comparison of functional outcome 1 year after minimally invasive plate osteosynthesis (MIPO) versus open reduction internal fixation (ORIF).* Eur J Trauma Emerg Surg, 2021.
15. Cai, M., et al., *Internal fixation versus shoulder hemiarthroplasty for displaced 4-part proximal humeral fractures in elderly patients.* Orthopedics, 2012. **35**(9): p. e1340-6.
16. Çaliskan, E. and Ö. Doğan, *PHILOS plate versus nonoperative treatment in 2-, 3-, and 4-part proximal humeral fractures: Comparison with healthy control subjects.* J Orthop Surg (Hong Kong), 2019. **27**(3): p. 2309499019875169.
17. Cha, S.M., H.D. Shin, and S.J. Hwang, *Temporary ipsilateral stiff shoulder after operative fixation of distal radial fractures.* J Shoulder Elbow Surg, 2017. **26**(6): p. 923-930.

18. Chen, C., et al., *[Clinical outcomes of open reduction and internal fixation in treating Gustilo typeI and II patients with open distal humeral fractures].* Zhongguo Gu Shang, 2019. **32**(4): p. 350-354.
19. Chen, C.Y., et al., *Preliminary clinical and radiographic outcomes of proximal humeral fractures: comparison of ALPS and PHILOS plating in Asian patients in Taiwan.* J Orthop Surg Res, 2020. **15**(1): p. 364.
20. Cho, C.H., D.H. Kim, and B.S. Kim, *Radiographic and clinical results of tension suture fixation using two washers with PHILOS plate for proximal humeral fractures.* Injury, 2017. **48**(2): p. 464-468.
21. Cohen, M., et al., *OSTEOSYNTHESIS OF PROXIMAL HUMERAL END FRACTURES WITH FIXED-ANGLE PLATE AND LOCKING SCREWS: TECHNIQUE AND RESULTS.* Rev Bras Ortop, 2009. **44**(2): p. 106-11.
22. Davids, S., et al., *Comparison of Locked Plating of Varus Displaced Proximal Humeral Fractures With and Without Fibula Allograft Augmentation.* J Orthop Trauma, 2020. **34**(4): p. 186-192.
23. Doshi, C., et al., *Treatment of Proximal Humerus Fractures using PHILOS Plate.* J Clin Diagn Res, 2017. **11**(7): p. Rc10-rc13.
24. Erasmo, R., G. Guerra, and L. Guerra, *Fractures and fracture-dislocations of the proximal humerus: A retrospective analysis of 82 cases treated with the Philos(®) locking plate.* Injury, 2014. **45 Suppl 6**: p. S43-8.
25. Falez, F., et al., *Low complication rates in Minimally Invasive Plate Osteosynthesis (MIPO) for proximal humeral fractures at 5 years of follow-up.* Injury, 2019. **50 Suppl 2**: p. S34-s39.
26. Faraj, D., et al., *Results of 131 consecutive operated patients with a displaced proximal humerus fracture: an analysis with more than two years follow-up.* Eur J Orthop Surg Traumatol, 2011. **21**(1): p. 7-12.
27. Fattoretto, D., A. Borgo, and C. Iacobellis, *The treatment of complex proximal humeral fractures: analysis of the results of 55 cases treated with PHILOS plate.* Musculoskelet Surg, 2016. **100**(2): p. 109-14.
28. Fazal, M.A. and F.S. Haddad, *Philos plate fixation for displaced proximal humeral fractures.* J Orthop Surg (Hong Kong), 2009. **17**(1): p. 15-8.
29. Fraser, A.N., et al., *Reverse Shoulder Arthroplasty Is Superior to Plate Fixation at 2 Years for Displaced Proximal Humeral Fractures in the Elderly: A Multicenter Randomized Controlled Trial.* J Bone Joint Surg Am, 2020. **102**(6): p. 477-485.
30. Geiger, E.V., et al., *Functional outcome and complications following PHILOS plate fixation in proximal humeral fractures.* Acta Orthop Traumatol Turc, 2010. **44**(1): p. 1-6.
31. George, P.K., et al., *Functional Outcome and Complications in Management of Proximal Humerus Fractures Operated with Proximal Humerus Locking Plate.* Malays Orthop J, 2021. **15**(2): p. 47-54.
32. Gönç, U., et al., *Minimally invasive plate osteosynthesis with PHILOS plate for proximal humerus fractures.* Acta Orthop Traumatol Turc, 2017. **51**(1): p. 17-22.
33. Gracitelli, M.E.C., et al., *Outcomes evaluation of locking plate osteosynthesis in displaced fractures of the proximal humerus.* Rev Bras Ortop, 2013. **48**(6): p. 491-499.
34. Gracitelli, M.E., et al., *Locking intramedullary nails compared with locking plates for two- and three-part proximal humeral surgical neck fractures: a randomized controlled trial.* J Shoulder Elbow Surg, 2016. **25**(5): p. 695-703.
35. Handschin, A.E., et al., *Functional results of angular-stable plate fixation in displaced proximal humeral fractures.* Injury, 2008. **39**(3): p. 306-13.

36. Hengg, C., et al., *Cement augmentation of the proximal humerus internal locking system in elderly patients: a multicenter randomized controlled trial.* Arch Orthop Trauma Surg, 2019. **139**(7): p. 927-942.
37. Jaura, G., J. Sikdar, and S. Singh, *Long Term Results of PHILOS Plating and Percutaneous K-Wire Fixation in Proximal Humerus Fractures in The Elderly.* Malays Orthop J, 2014. **8**(1): p. 4-7.
38. Klitscher, D., et al., *Osteosynthesis of Proximal Humeral Fractures with the Fixed Angle PHILOS-plate.* Eur J Trauma Emerg Surg, 2008. **34**(1): p. 29-36.
39. Koukakis, A., et al., *Fixation of proximal humerus fractures using the PHILOS plate: early experience.* Clin Orthop Relat Res, 2006. **442**: p. 115-20.
40. Kumar, G.N., et al., *Surgical treatment of proximal humerus fractures using PHILOS plate.* Chin J Traumatol, 2014. **17**(5): p. 279-84.
41. Launonen, A.P., et al., *Operative versus non-operative treatment for 2-part proximal humerus fracture: A multicenter randomized controlled trial.* PLoS Med, 2019. **16**(7): p. e1002855.
42. Lee, W., J.Y. Park, and Y.M. Chun, *Operative Treatment of 2-Part Surgical Neck Fracture of the Humerus: Intramedullary Nail Versus Locking Compression Plate With Technical Consideration.* J Orthop Trauma, 2017. **31**(9): p. e270-e274.
43. Leonard, M., et al., *The use of locking plates in proximal humeral fractures: Comparison of outcome by patient age and fracture pattern.* Int J Shoulder Surg, 2009. **3**(4): p. 85-9.
44. Lorenz, G., et al., *Complication rate after operative treatment of three- and four-part fractures of the proximal humerus: locking plate osteosynthesis versus proximal humeral nail.* Eur J Trauma Emerg Surg, 2021. **47**(6): p. 2055-2064.
45. Luciani, P., et al., *Angular stable plate versus reverse shoulder arthroplasty for proximal humeral fractures in elderly patient.* Musculoskelet Surg, 2022. **106**(1): p. 43-48.
46. Martinez, A.A., J. Cuenca, and A. Herrera, *Philos plate fixation for proximal humeral fractures.* J Orthop Surg (Hong Kong), 2009. **17**(1): p. 10-4.
47. Matejcić, A., et al., *Internal fixation with locking plate of 3- and 4-part proximal humeral fractures in elderly patients: complications and functional outcome.* Acta Clin Croat, 2013. **52**(1): p. 17-22.
48. Miyazaki, A.N., et al., *EVALUATION OF THE COMPLICATIONS OF SURGICAL TREATMENT OF FRACTURES OF THE PROXIMAL EXTREMITY OF THE HUMERUS USING A LOCKING PLATE.* Rev Bras Ortop, 2012. **47**(5): p. 568-74.
49. Moonot, P., N. Ashwood, and M. Hamlet, *Early results for treatment of three- and four-part fractures of the proximal humerus using the PHILOS plate system.* J Bone Joint Surg Br, 2007. **89**(9): p. 1206-9.
50. Norouzi, M., et al., *Clinical results of using the proximal humeral internal locking system plate for internal fixation of displaced proximal humeral fractures.* Am J Orthop (Belle Mead NJ), 2012. **41**(5): p. E64-8.
51. Ockert, B., et al., *Position of polyaxial versus monoaxial screws in locked plating for proximal humeral fractures: analysis of a prospective randomized study.* Eur J Orthop Surg Traumatol, 2014. **24**(5): p. 747-52.
52. Oh, H.K., et al., *Lessons learned from treating patients with unstable multifragmentary fractures of the proximal humerus by minimal invasive plate osteosynthesis.* Arch Orthop Trauma Surg, 2015. **135**(2): p. 235-242.
53. Olerud, P., et al., *Quality of life and functional outcome after a 2-part proximal humeral fracture: a prospective cohort study on 50 patients treated with a locking plate.* J Shoulder Elbow Surg, 2010. **19**(6): p. 814-22.

54. Ortmaier, R., et al., *Comparison between minimally invasive, percutaneous osteosynthesis and locking plate osteosynthesis in 3-and 4-part proximal humerus fractures.* BMC Musculoskelet Disord, 2015. **16**: p. 297.
55. Papadopoulos, P., et al., *Mid-term results of internal fixation of proximal humeral fractures with the Philos plate.* Injury, 2009. **40**(12): p. 1292-6.
56. Parmaksizoğlu, A.S., et al., *Locking plate fixation of three- and four-part proximal humeral fractures.* Acta Orthop Traumatol Turc, 2010. **44**(2): p. 97-104.
57. Plath, J.E., et al., *Locking nail versus locking plate for proximal humeral fracture fixation in an elderly population: a prospective randomised controlled trial.* BMC Musculoskelet Disord, 2019. **20**(1): p. 20.
58. Robinson, C.M., et al., *Proximal humeral fractures with a severe varus deformity treated by fixation with a locking plate.* J Bone Joint Surg Br, 2010. **92**(5): p. 672-8.
59. Seo, J.B., et al., *Assessment of the efficacy of the far cortical locking technique in proximal humeral fractures: a comparison with the conventional bi-cortical locking technique.* BMC Musculoskelet Disord, 2020. **21**(1): p. 800.
60. Setaro, N., et al., *Surgical management of 2- or 3-part proximal humeral fractures: comparison of plate, nail and K-wires.* Musculoskelet Surg, 2022. **106**(2): p. 163-167.
61. Shahid, R., et al., *Outcome of proximal humerus fractures treated by PHILOS plate internal fixation. Experience of a district general hospital.* Acta Orthop Belg, 2008. **74**(5): p. 602-8.
62. Shi, H.F., et al., *Management of proximal humeral fractures in elderly patients with uni- or polyaxial locking osteosynthesis system.* Arch Orthop Trauma Surg, 2011. **131**(4): p. 541-7.
63. Shin, W.C., et al., *High bone union rate using a locking plate for proximal humeral fractures in patients older than 70 years: importance of the medial column.* Eur J Trauma Emerg Surg, 2021.
64. Siebenbürger, G., et al., *Screw-tip augmentation versus standard locked plating of displaced proximal humeral fractures: a retrospective comparative cohort study.* J Shoulder Elbow Surg, 2019. **28**(7): p. 1326-1333.
65. Sohn, H.S., et al., *Clinical comparison between open plating and minimally invasive plate osteosynthesis for displaced proximal humeral fractures: A prospective randomized controlled trial.* Injury, 2017. **48**(6): p. 1175-1182.
66. Spross, C., et al., *Surgical treatment of Neer Group VI proximal humeral fractures: retrospective comparison of PHILOS® and hemiarthroplasty.* Clin Orthop Relat Res, 2012. **470**(7): p. 2035-42.
67. Spross, C., et al., *The PHILOS plate for proximal humeral fractures--risk factors for complications at one year.* J Trauma Acute Care Surg, 2012. **72**(3): p. 783-92.
68. Urda, A., et al., *Management of displaced surgical neck fractures of the humerus: health related quality of life, functional and radiographic results.* Injury, 2012. **43 Suppl 2**: p. S12-9.
69. Vijayvargiya, M., A. Pathak, and S. Gaur, *Outcome Analysis of Locking Plate Fixation in Proximal Humerus Fracture.* J Clin Diagn Res, 2016. **10**(8): p. Rc01-5.
70. Voigt, C., et al., *Are polyaxially locked screws advantageous in the plate osteosynthesis of proximal humeral fractures in the elderly? A prospective randomized clinical observational study.* J Orthop Trauma, 2011. **25**(10): p. 596-602.
71. Wang, H., et al., *Locking Plate Use with or without Strut Support for Varus Displaced Proximal Humeral Fractures in Elderly Patients.* JB JS Open Access, 2019. **4**(3).
72. Xue, G., et al., *Titanium mini locking plate with trans-osseous sutures for the treatment of humeral greater tuberosity fracture osteosynthesis versus PHILOS: a retrospective view.* Int Orthop, 2018. **42**(10): p. 2467-2473.

73. Zeng, L.Q., et al., *Influence of Medial Support Screws on the Maintenance of Fracture Reduction after Locked Plating of Proximal Humerus Fractures.* Chin Med J (Engl), 2018. **131**(15): p. 1827-1833.
74. Zhao, L., et al., *Comparison of the Effects of Proximal Humeral Internal Locking System (PHILOS) Alone and PHILOS Combined with Fibular Allograft in the Treatment of Neer Three- or Four-part Proximal Humerus Fractures in the Elderly.* Orthop Surg, 2019. **11**(6): p. 1003-1012.
